# Aging and Alzheimer’s: the critical role of mitochondrial dysfunction and synaptic alterations

**DOI:** 10.3389/fnsyn.2025.1676317

**Published:** 2026-01-02

**Authors:** Pinky, Zitin Wali, Neha, Prachi Tiwari, Mohamed El-Tanani, Syed Arman Rabbani, Suhel Parvez

**Affiliations:** 1 Department of Toxicology, School of Chemical and Life Sciences, Jamia Hamdard, New Delhi, India; 2 Department of Physiotherapy, School of Nursing Sciences and Allied Health, Jamia Hamdard, New Delhi, India; 3 RAK College of Pharmacy, RAK Medical and Health Sciences University, Ras Al Khaimah, United Arab Emirates; 4 Department of Clinical Pharmacy and Pharmacology, RAK College of Pharmacy, RAK Medical and Health Sciences University, Ras Al Khaimah, United Arab Emirates

**Keywords:** aging, Alzheimer’s disease, bioenergetics, synaptic plasticity, mitochondrial free radical production and longevity

## Abstract

Alzheimer’s disease is a progressive neurodegenerative disorder marked by cognitive decline, accumulation of amyloid-β plaques and neurofibrillary tangles, synaptic dysfunction, and mitochondrial impairment. Despite multiple therapeutic strategies, currently available treatments only provide symptomatic relief without halting disease progression. Emerging evidence implicates mitochondrial dysfunction–including oxidative stress, impaired calcium signaling, mitophagy deficits, disrupted proteostasis, and electron transport chain abnormalities, as central to AD pathogenesis. These dysfunctions contribute to synaptic degeneration, increased reactive oxygen species, and neuronal death. This review consolidates current knowledge on the mechanistic pathways of mitochondrial impairment in AD and their downstream effects on neuronal health. We also explore the therapeutic potential of multitarget approaches, including agents targeting Aβ and tau pathology, oxidative stress mitigation, mitochondrial quality control, and synaptic restoration. By integrating evidence from recent preclinical and clinical studies, this work highlights mitochondrial homeostasis as a promising frontier for disease-modifying therapies in AD.

## Graphical abstract

This illustration describes how mitochondrial dysfunction acts as a central driver of Alzheimer’s disease (AD) pathology. Intramitochondrial accumulation of amyloid-β (aβ) and hyperphosphorylated tau disrupts the electron transport system (ETS), leading to reduced ATP production and excessive reactive oxygen species (ROS) generation. Elevated ROS, impaired mitophagy, and proteostasis failure exacerbate oxidative damage, while calcium (Ca^2+^) dyshomeostasis amplifies mitochondrial stress. Together, these events precipitate a bioenergetic crisis and synaptic degeneration, ultimately contributing to neuronal loss and cognitive decline in AD.
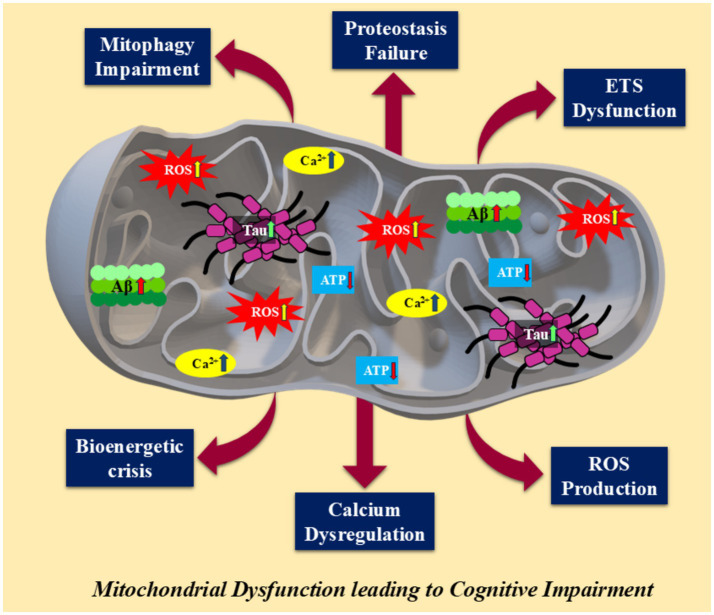

## 1 Introduction

Alzheimer’s disease (AD) is a degenerative brain disorder that is characterized by memory loss and the accumulation of two insoluble protein clumps, i.e., amyloid beta (Aβ) plaques and tau neurofibrillary tangles (NFTs). As of 2024, more than 55 million people worldwide are living with AD and other dementias, with nearly 10 million new cases emerging each year. This number is projected to rise sharply, reaching an estimated 139 million by 2050, highlighting the escalating global public health burden of AD (Alzheimer and Association, 2024). Dementia is a neurological illness that impedes a person’s ability to live a fully functional and independent life by compromising many cognitive areas. The prevalence of AD climbs exponentially with age, from 3% to 32% between the ages of 65 and 85 (Haque and Levey, 2019).

Multiple years of research have indicated that mitochondrial respiratory complex dysfunction has long been associated with the aetiology of neurodegenerative diseases such as AD. The finding of impaired oxygen and glucose transport in the brains of AD patients is the most significant indirect evidence supporting mitochondrial participation in the disease. According to the mitochondrial cascade theory, the other clinical symptoms of AD should be considered side effects, as mitochondrial malfunction is the primary cause in the majority of instances (Swerdlow, 2018; Swerdlow and Khan, 2004). Electron microscope scans of the brains of AD patients have revealed altered mitochondrial morphology, including smaller mitochondria, altered and broken cristae, accumulation of osmophilic components, lipofuscin vacuoles, and elongated connected organelles. Numerous studies have been undertaken to evaluate the relationship between alterations in mitochondria (mtDNA) and AD, which have demonstrated that mtDNA levels in the brain cells and cerebrospinal fluid of AD patients have been reduced (Monzio Compagnoni et al., 2020; Swerdlow, 2018; Wei et al., 2017).

Oxidative phosphorylation (OXPHOS), which serves as the cell’s energy source, produces the majority of the adenosine triphosphate (ATP). Neurons are the most ATP-consuming cell type. The primary reason for this is the requirement to maintain the ionic gradients required for ongoing neurotransmission, electrophysiological activity, and transient synaptic plasticity. In addition to being significant sources of free radical generation, defective mitochondria can trigger apoptosis by releasing cytosolic cytochrome C (cyt). Consequently, neuronal damage could result from even a little reduction in mitochondrial function (Sharma et al., 2021). Significant mitochondrial dysfunction also occurs, and there are variations in the number, ultrastructure, and enzyme activity of mitochondria in people with AD. The additional symptoms of AD are likely a result or cause of mitochondrial malfunction. The integrated stress response (ISR), autophagy/mitophagy, proteosome activity, and the mitochondrial unfolded protein response (mtUPR) are examples of pertinent retrograde reactions (Weidling and Swerdlow, 2020).

The pathogenesis of AD has been explained through several competing and overlapping models, including the amyloid cascade, tau-first, and mitochondrial cascade hypotheses. While the amyloid and tau models emphasize extracellular plaque and cytoskeletal pathology, respectively, accumulating evidence suggests that mitochondrial dysfunction may act as an upstream trigger influencing both Aβ aggregation and tau hyperphosphorylation (Swerdlow, 2018, 2023; Wang et al., 2020). Mitochondrial impairments disrupt calcium buffering, redox balance, and neurotransmission, thereby coupling energy failure with synaptic loss (Wong et al., 2020). Furthermore, defective mitophagy and impaired mitochondrial quality control have emerged as converging mechanisms linking oxidative stress to proteinopathy (Mary et al., 2023). This review extends prior syntheses by integrating mitochondrial bioenergetics, calcium signaling, and synaptic resilience into a unified framework of AD progression. In contrast to previous reviews, we critically evaluate these interconnections and identify therapeutic targets that restore mitochondrial–synaptic homeostasis (Sheng and Cai, 2012).

## 2 Pathophysiology of AD

AD is characterized by the accumulation of Aβ proteins on the extracellular layer of neurons and the formation of NFTs as a result of the accumulation of intracellular tau proteins owing to hyperphosphorylation. A deficiency of the neurotransmitter acetylcholine and oxidative stress caused by increased glutamatergic transmission are also associated with AD. AD is typically the leading cause of dementia in elderly people when the course of symptoms becomes chronic. In the early stages of AD, memory losses are distinct, the limbic system’s cholinergic neurons are affected, and the hippocampus volume falls by around 25%. The second stage, characterized by difficulty in recognizing and interacting with individuals, is a protracted stage and can last from 2 to 10 years; it is related to damaged neurons since they are in charge of short- and long-term memory (dos Santos Picanco et al., 2016). The primary medical feature is dementia, which is intended to be the result of memory loss and executive dysfunction that interfere with day-to-day functioning. It is also conceivable for atypical presentations to be characterized by a deficit in other domains, such as linguistic, visual, or executive abilities (Scheltens et al., 2016).

### 2.1 Mechanistic overview of Aβ aggregation and tau hyperphosphorylation

#### 2.1.1 Aβ aggregation

Aβ is a 4 kDa portion of the larger parent molecule, amyloid precursor protein (APP), which is widely synthesized by blood cells, particularly platelets, and astrocytes. Aβ is produced as a result of two further proteolytic divisions of APP by the enzymes β- and *γ*-secretase and beta-APP-cleaving enzyme-1 (BACE1), respectively, at the ectodomain and intra-membranous locations (Hampel et al., 2021). The considerably bigger APP is sequentially cleaved by proteases β- and γ-secretase to produce Aβ. In contrast, Aβ formation is prevented by the breakdown of APP by *α*-secretase. Aβ takes on a highly organized shape known as a cross—β spine, or amyloid, in the AD brain (Zhang et al., 2018). For the development of synapses, dendritic spines, and synaptic activity, APP is required, aiding in the retention of information and memory (Rajmohan and Reddy, 2017). On chromosome (Chr) 21, APP is what creates Aβ, leading to its buildup in neocortical areas. Individuals with Down syndrome, a genetic disorder, are impacted by the APP gene’s location on Chr 21 since their trisomy increases their risk of Aβ aggregate formation and early onset of AD (EOAD) (Dorostkar et al., 2015; John and Reddy, 2021). Additionally, due to decreased activity, *γ*-secretase do not cleave APP c-terminal segments. One of the numerous potential reasons for AD is the buildup of APP c-terminal segments and decreased APP metabolism (Kametani and Hasegawa, 2018). Chemical modifications of Aβ are key markers of AD and potential therapeutic targets, though the exact molecular functions and dynamics leading to synaptic breakdown and dementia are still intensely studied (Hampel et al., 2021; McDade and Bateman, 2017). Both extracellularly and intracellularly, Aβ can be detected. In APP models, intracellular amyloid buildup rises with age and has been linked to spine loss (John and Reddy, 2021). The receptor for advanced glycosylation end products (RAGE) can absorb extracellular Aβ into neurons (Dorostkar et al., 2015). Aβ acquires an extremely organized form referred to as amyloid or cross-spine. Three phases, which include the nucleation, elongation, and stationary phases, can be distinguished in the development of Aβ plaques or fibrils (Iadanza et al., 2018).

**Figure 1:**
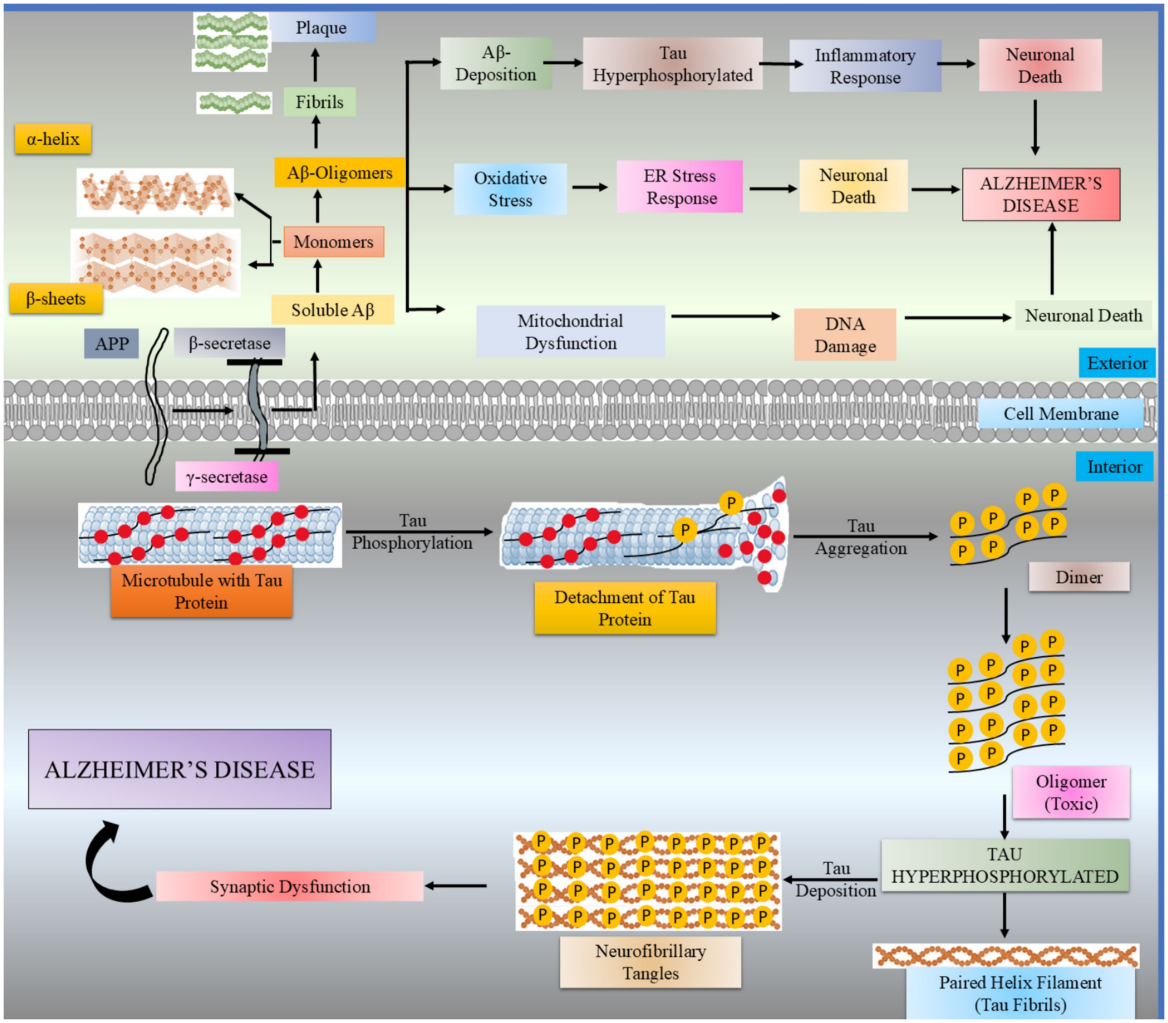
AD is thought to be caused by a pathophysiological cascade involving the interplay between tau hyperphosphorylation and amyloid beta.

Oligomeric Aβ produces a nucleus during the nucleation phase that may result in numerous monomers. As fibrils expand, they may break, giving rise to new aggregation-prone species that extend the fibril. A wide range of insoluble fibres, oligomers, and soluble monomers establish a stable equilibrium in the stationary state until all free monomers are transformed into fibrillar forms. In comparison to fully developed fibres, oligomers are thought to be harmful. It is unclear that Aβ assemblies are the most harmful, though in order to create the plagues, the fibres also interact with one another alongside additional proteins as well as non-proteinaceous components (Stewart et al., 2017; Zhang et al., 2018). The APP gene was ultimately sequenced, confirming that Aβ is a byproduct of APP’s enzymatic activity. Eventually, abundant Aβ aggregates were identified as the primary component of neocortical neuritic plaques, a clinical marker for AD. Memory and motor deterioration associated with AD across the disease progression from preclinical to prodromal and cognitive stages is caused by the spatiotemporal progression of these pathophysiological changes (Hampel et al., 2021). Research using biomarkers that were carried out in EOAD and late onset of AD (LOAD) has revealed a chronological sequence between the early stages of Aβ pathology, the development of Aβ aggregation species and plaques across the brain, and ultimately tau and natural signatures associated with neurodegeneration. Although no direct correlation has been found between Aβ pathology and AD-related physiological modifications occurring across multiple temporal dimensions, a growing corpus of clinical and animal studies indicates that Aβ aggregation species may facilitate further pathophysiological advancement and/or evolve alongside them (Jack et al., 2018; Sperling et al., 2020; Tachibana et al., 2019) (Figure 1).

#### 2.1.2 Tau hyperphosphorylation

The protein tau that attaches to microtubules (MTs) plays a role in controlling the processes of axon expansion and axonal flow by helping to keep axonal MTs stable. Post-translational alterations, primarily phosphorylation, which also regulates a number of other lesser-known tau functions, influence tau’s ability to bind to MTs (Sotiropoulos et al., 2017). NFTs, a clinical marker present in the brains of people with AD, were shown to include an essential element known as tau (Grundke-Iqbal et al., 1986; Iqbal et al., 2016). Implicitly, the progression of AD and other neurological disorders has since been based on the gradual pathological deposition of phosphorylated and aggregated tau (Iqbal et al., 2016). It was determined that tau hyperphosphorylation is connected to tau accumulation and toxicity because accumulated and soluble tau from brains with AD reacts with both antibodies that identify phosphorylated and non-phosphorylated tau following its alkaline phosphatase digestion (Augustinack et al., 2002; Wegmann et al., 2021a). The tau protein bands are noticeably pushed toward a large molecular mass in SDS-PAGE gels, demonstrating strong phosphorylation of tau isolated from the AD brain. Healthy adult brains, on the other hand, exhibit a tau protein that appears to be in an insufficiently phosphorylated phase (Rawat et al., 2022).

In both primary and secondary tauopathies, an aberrant buildup of cyclooxy-tau in disease morphological inclusions serves as a clinal marker (Iqbal et al., 2010). Tau pathology begins in two separate brain regions (entorhinal cortex and hippocampus) in AD and then expands in a predetermined arrangement of spaces across the rest of the brain (Wegmann et al., 2021a). Tau aggregate assembly and shape, as well as their capacity to serve as seeds for tau aggregation, might be affected by the alteration of tau in aggregates from AD brains (Arakhamia et al., 2020). Preclinical treatment strategies that minimize the percentage of overall tau in adults’ brains on an mRNA level do not require knowledge of a precise “toxic tau” target (DeVos et al., 2013, 2017; Wegmann et al., 2021b). The central nervous system (CNS) neuronal axons have the highest concentration of tau, which has been observed in oligodendrocytes, non-neural tissues, and the somatodendritic sections of the neurons (Šimić et al., 2016). Abnormal changes in tau protein are believed to cause hyperphosphorylation, leading to the formation of helical filaments and NFTs in AD and other tauopathies (Sinsky et al., 2021; Reddy, 2011). One of the main pathogenic characteristics of AD and other tauopathies is the accumulation of tau in the neutritis and neural cells, where it forms NFTs after becoming separated from MTs (Reddy and Reddy, 2011). The fact that frontotemporal dementia and Parkinsonism are associated with tau gene mutations suggests that tau malfunction may contribute to neurodegeneration.

According to increasing research, oxidative stress may be one of the causes of tauopathies because it is crucial for tau hyperphosphorylation, polymerization, and toxicity (Alavi Naini and Soussi-Yanicostas, 2015; da Costa et al., 2022). Tau proteins interact with many different molecules in the crowded cytosol because they are relatively flexible, highly soluble, and contain numerous charges (Lim et al., 2014; Bhatia and Sharma, 2021). Reactive oxygen species (ROS), which are the primary source of oxidative stress and are created in excess by mitochondria, peroxisomes, and endoplasmic reticulum (Alavi Naini and Soussi-Yanicostas, 2015). Numerous mechanisms, including mitochondrial dysfunction, that result in electron transport chain (ETC) deficiencies and the generation of ROS contribute to their accumulation in AD (Bhatia and Sharma, 2021). The aberrant phosphorylation of tau in AD leads to a breakdown of synapses and neurons in the CNS. At least a threefold rise in the phosphorylation of tau characterizes this in brains with AD (Neddens et al., 2018). Hyperphosphorylated tau can be classified into three types: AD tau, which closely resembles unhybridized tau; AD phosphorylated tau, which is soluble; and paired helical filaments (PHFs), which contain insoluble tau (Alonso et al., 2018). The accumulation of tau impairs neuronal function, although the precise mechanism of tau-induced cytotoxicity remains unclear. Hyperphosphorylation at multiple sites causes tau to detach from MTs and disrupt intracellular transport, leading to reduced MT binding in AD and contributing to neuronal death (Figure 1) (Kanaan et al., 2013).

## 3 Signatures of AD and its association with synaptic plasticity

The progression of synapse failure, neuronal cell death, and mitochondrial dysfunction have all been strongly linked to AD. Despite years of study, AD still holds a lot of mysteries behind these hallmarks. A greater understanding of disease pathogenesis and progression is the goal of research. The hopes of those conducting research in this area of study are to find and treat the causes of this disease, or better yet, to prevent it altogether. The pace at which free radicals are generated in mitochondria leads to various adaptive responses and subsequent molecular damage to cellular components. This includes damage to mitochondrial DNA, which has significant biological implications that impact the ageing process in different animal species. Among all cell types, neurons use some of the most ATP. This is mostly due to the necessity to maintain the ionic gradients required for ongoing electrical and physiological activity, neuronal transmission, and transient synaptic plasticity. The environment or a person’s experience can influence the strength and effectiveness of neuronal connections and interactions. This property, known as synaptic plasticity, was first characterized at the cellular level and is intimately related to learning and memory processes (Cuestas Torres and Cardenas, 2020). MicroRNAs (miRNAs) are interestingly recognized as essential mediators of synaptic plasticity. Synapse creation and synaptic plasticity are governed by modifications in brain miRNA expression that modify dendritic spine morphology or local protein expression that contributes to the transmission of synaptic information, both of which influence synaptic function (Reza-Zaldivar et al., 2020). It is now known that synaptic plasticity is the mechanism by which synapses change their molecular makeup and structural characteristics in response to particular types of brain activity (Frere and Slutsky, 2018; Oby et al., 2019). Research has demonstrated that neural network-altering events, such as long-term depression (LTD) and long-term potentiation (LTP), alter the structure and function of synapses, which in turn affect synaptic plasticity (Ju and Zhou, 2018).

**Figure 2:**
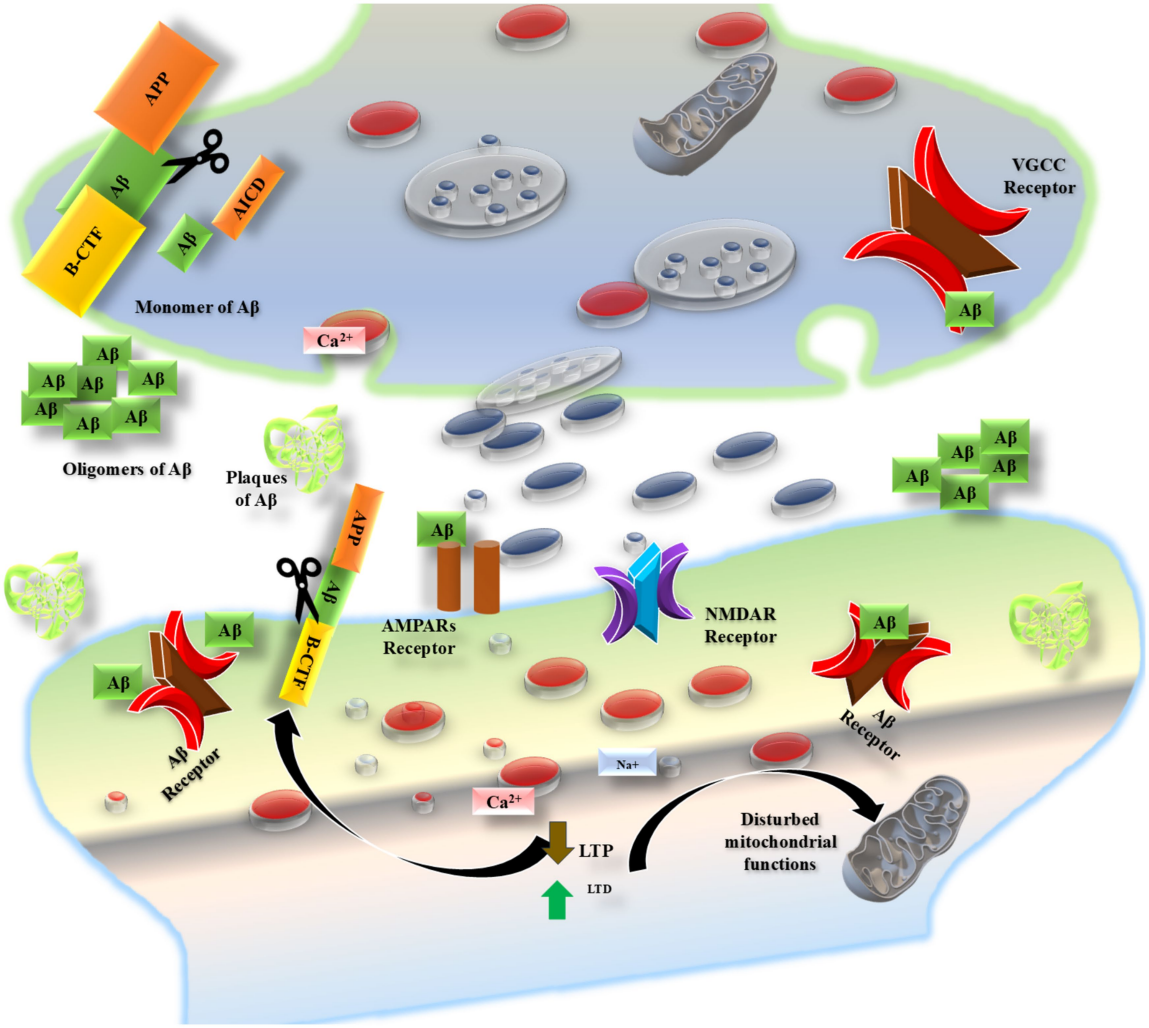
The cognitive impairment associated with AD is correlated with synaptic loss. An “AD synapse” encapsulates the documented discoveries about the impact of Aβ buildup on synaptic plasticity mechanisms. BACE1 and *γ*-secretase process APP, leading to a rise in Aβ concentration. The disruption of Ca^2+^ homeostasis resulting from these elevated levels of Aβ occurs via multiple pathways: (1) an increase in calcium release via InsP3Rs and RyR receptors from the stores in RE; (2) irregular Ca^2+^ ion flow through NMDARs because ADDLs bind to these receptors; (3) formation of pores permeable to Ca^2+^ in the plasma membrane; and (4) induction of endocytosis of AMPARs with GluA2/GluA3.

Synaptic plasticity acts at the molecular level through dendritic spine transport, function, and structural variations, which include elongation, contraction, and morphological modifications (Chidambaram et al., 2019). According to research, Aβ-induced excitotoxicity changes electrical pathways in LTP and LTD and disrupts the balance between spine production and ejection adjacent to amyloid plaques, resulting in a significant loss of dendritic spines (Reza-Zaldivar et al., 2020). A growing body of evidence has suggested recently that mitochondria play a crucial and distinctive function in the transmission of synapses. Every step of neurotransmission, which includes the generation and preservation of neurotransmitters, the movement of synaptic vesicles (SVs), the discharge of neurotransmitters from presynaptic synapses, and the reuse of SVs, is thought to be regulated by mitochondria (Guo et al., 2017). A classic example of mitochondrial variation is found in neurons. Neuronal mitochondria are divided into numerous categories depending on their location and functionality. Among them, synaptic mitochondria are widely acknowledged to play a significant role in maintaining synaptic activity. It is widely recognized that mitochondria encourage the transmission of synaptic information, mainly through their roles in governing the synthesis of ROS, supplying energy, and sustaining calcium equilibrium, as well as by synthesizing critical intermediary substances or end products of a number of neurotransmitters. It is possible that synaptic function is impaired in diseased situations by mitochondrial deficiencies (Guo et al., 2017). Synaptic mitochondria serve as essential regulators of neuronal communication by coupling bioenergetic and signaling demands at pre- and postsynaptic terminals. They supply ATP for vesicle recycling, receptor trafficking, and actin remodeling, while buffering calcium transients to prevent excitotoxicity and sustain long-term potentiation (Devine and Kittler, 2018; Walters and Usachev, 2023). Precise localization of mitochondria within dendritic spines further supports activity-dependent plasticity, as shown by their strategic positioning to modulate synaptic strength (Thomas et al., 2023). Disruption of mitochondrial trafficking and calcium handling impairs neurotransmission, leading to synaptic loss characteristic of Alzheimer’s disease (Mishra et al., 2025; Wang et al., 2023). Recent evidence highlights a bidirectional “mitochondrial–synaptic plasticity” crosstalk, whereby synaptic activity remodels mitochondrial dynamics and vice versa, integrating energy metabolism with structural and functional resilience (Sayehmiri et al., 2024) (Figure 2).

### 3.1 Synaptic plasticity modulation by fluid biomarkers

#### 3.1.1 Brain derived neurotrophic factor (BDNF)

BDNF plays an important role in synaptic plasticity, especially inside the hippocampus, which is critical for establishing memories and preserving the structural integrity of the brain (Coelho et al., 2014). In AD, BDNF plasma levels vary significantly among stages. The levels are initially higher in early stages of suspected AD, when compared with, severe AD and healthy controls. This may indicate a compensatory strategy for neurodegeneration (Allen et al., 2011). Recent research, however, shows a decline in BDNF levels in individuals with AD and mild cognitive impairment (MCI) (Borba et al., 2016). This decrease in BDNF is associated with lower performance on episodic memory tests like the Auditory Verbal Learning Test (Yu et al., 2008). Additionally, reduced levels of BDNF may precede obvious neuronal damage, since lower BDNF is associated with hippocampus contraction in AD and MCI patients. Therapeutic treatments also alter BDNF levels. For example, repeated transcranial magnetic stimulation (rTMS) has been demonstrated to raise BDNF and enhance cognitive abilities, like visual memory and clock-drawing test scores (Velioglu et al., 2021). This shows that BDNF might be a useful biomarker to track synaptic plasticity and the efficiency of treatments in AD. Also, reduced BDNF in cerebrospinal fluid (CSF) has been discovered to predict the transition from MCI to AD, suggesting that it may function in monitoring neurological disorders (Forlenza et al., 2015).

#### 3.1.2 Vascular endothelial growth factor (VEGF)

The VEGF-A gene encodes a critical protein involved in blood vessel formation, maintenance, oxygen, and nutrition delivery to the brain. VEGF is positively associated with cognitive retention in elderly people, primarily due to its role in neurogenesis ad synaptic plasticity (Lange et al., 2016; Tubi et al., 2021). In AD, amyloid-induced changes in VEGF signalling can impact cognitive abilities. Exogenous infusion of VEGF in AD mouse models has shown improvements in memory function and reduction in Aβ and tau loads (Religa et al., 2013). Although some studies have reported no substantial variations in VEGF concentration between AD patients and healthy controls, higher VEGF levels in CSF have been associated with slower cognitive decline, suggesting a potential neuroprotective role. However, other studies have shown that VEGF’s interactions with Aβ42 and t-tau correlate to memory deterioration in AD, indicating that VEGF could serve as a biomarker reflecting both synaptic plasticity and disease progression (Hohman et al., 2015).

#### 3.1.3 Omega 3 polyunsaturated fatty acids (PUFAs)

PUFAs, especially omega-3 FAs like docosahexaenoic acid (DHA) and eicosapentaenoic acid (EPA), are becoming recognized for their possible significance in synaptic plasticity and cognitive function in AD (Bartochowski et al., 2020; Hill et al., 2019). Although high DHA dosages failed significantly delay brain shrinkage in mild to severe AD throughout a 12-month clinical study, other studies have yielded encouraging outcomes (Lee et al., 2013). For example, DHA supplementation was linked to increased hippocampus volume in individuals with MCI after a year, demonstrating that it has the ability to improve synaptic health (Yurko-Mauro et al., 2010). DHA, EPA, Vit D and Resveratrol have been shown to improve Aβ phagocytosis by monocytes, indicating potential neuroprotective benefits. Omega-3 treatment raised plasma levels of transthyretin (TTR), a protein that binds Aβ and inhibits plaque formation, suggesting the ability to serve as a biomarker (Faxén-Irving et al., 2013).

## 4 Regulatory mechanism of mitochondrial bioenergetics

**Figure 3:**
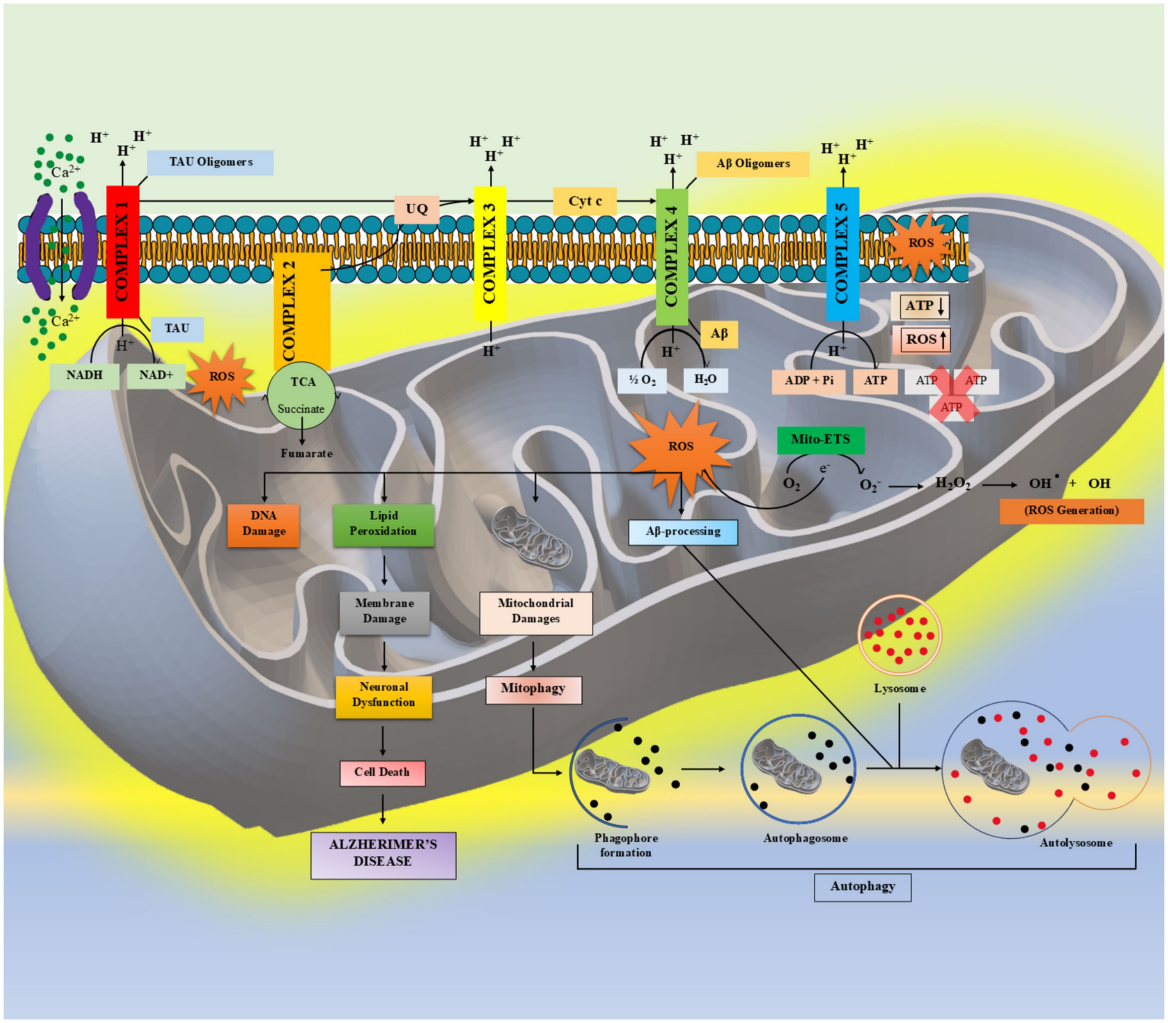
A schematic representation of the interplay between mitochondrial free radical generation and their enzymatic antioxidant defences. The production of ROS within mitochondria is a contributing factor to the development of AD. The presence of a malfunction in either complex I to IV within the mitochondrial respiratory chain facilitates the buildup of reactive radicals, thereby causing oxidative stress.

Mitochondria, the double-membrane organelles present in nearly all mammalian cells, are central to energy metabolism and neuronal survival (Youle and Van Der Bliek, 2012). Their morphology and distribution dynamically adapt to metabolic stress and cellular energy demands (Friedman and Nunnari, 2014). Through the electron transport chain (ETC), complexes I–IV establish a proton gradient that drives ATP synthesis via F₀F₁-ATP synthase (Complex V) (Cadonic et al., 2016). In AD, deficits in ETC activity, particularly within complexes I-IV, have been reported in the neocortex, hippocampus, and platelets, indicating systemic mitochondrial dysfunction (Guo et al., 2017; Beck et al., 2016; Du et al., 2011). Dysregulation of complex V in synaptic mitochondria further impairs bioenergetics and neuronal communication (Guo et al., 2017). In parallel, calcium (Ca^2+^) signaling, a critical regulator of neuronal excitability and mitochondrial bioenergetics, becomes severely disrupted in AD. Excessive Ca^2+^ influx through NMDA receptors, Aβ-induced pore formation, and abnormal release from intracellular stores cause cytosolic Ca^2+^ overload, leading to mitochondrial depolarization, ROS generation, and apoptotic signaling (Calvo-Rodriguez and Bacskai, 2021; Chami, 2021; Bagheri-Mohammadi et al., 2023). Because mitochondria both buffer and regulate Ca^2+^, their dysfunction amplifies this imbalance, driving oxidative stress and synaptic failure (Raghav et al., 2024). Moreover, impaired Ca^2+^ homeostasis intersects with defective autophagy and proteostasis, accelerating the accumulation of dysfunctional mitochondria (Hadi et al., 2024; Joshi et al., 2023). Therapeutic strategies targeting mitochondrial Ca^2+^ uptake and homeostasis show promise in restoring neuronal function and delaying AD progression (Song et al., 2024). Together, mitochondrial respiratory chain deficiencies and Ca^2+^ dysregulation represent converging mechanisms that fuel the bioenergetic crisis and neurodegeneration in AD (Figure 3).

### 4.1 Complex II

Complex II is thought to fit amid complexes III and IV, which together make up the megacomplex formed by complexes I, III, and IV. Two replicas from every complex make up each megacomplex. In extremely twisted regions of cristae, complex V, which does not belong to any mega complexes, seems to generate arrays of V-shaped dimers (He et al., 2018). Krebs cycle, glycolysis, and, to a lesser extent, acetyl-CoA transformation has decreased NADH molecules, which have become available to pass through ETC. This structure is made up of a series of reducing agents’ that are primarily divided into four primary protein-metal complexes, which use the movement of electrons to fuel the synthesis of ATP. The ETC is made of four essential protein complexes: complex I, II, III and IV of the coenzyme Q oxidoreductase family are NADH-coenzyme Q oxidoreductase, succinate-coenzyme Q oxidoreductase, and cyt C oxidate. ATP synthase, also called complex V, is another essential enzyme involved in OXPHOS. It also produces ATP as the outcome of electron circulation via ETC. Complex V is dispersed among several complexes of proteins that are found on the inner layer of the mitochondrial membrane that divides the interior matrix of mitochondria from the inter-mitochondrial gap (Cadonic et al., 2016). Forty five protein subunits make up complex I, which is divided into a short hydrophilic section and a long hydrophobic section, which is exclusively located in the interior of the membrane (Scheffler, 2007). Since the short section of complex I comprise NADH dehydrogenase, the flavin mononucleotide (FMN) cofactor, and the NADH binding site, each of which is required for NADH oxidation and consequently electron distribution into the system, this section starts with the procedure for the flow of electrons in ETC. The FMN cofactor serves as a relay centre by dividing the two electrons amongst the chain of seven Fe-S groups for transmission and holding an Fe-S group while NADH dehydrogenase oxidizes NADH by absorbing two electrons from it. One electron moves across the complex at a time as an outcome of the splitting mechanism. The small component of complex one does this by transferring ions from NADH across the complex into a membrane-bound electron transporter, ubiquinone (Q), which is then reduced to ubiquinol (OH_2_). Protein relocation is carried out by the long-chain component of complex one during this process. Although the precise process is unknown, it is believed that a pair of protons are transported via a redox-driven Q-cycle-related mechanism and two protons are transported via structural alteration (Cadonic et al., 2016; Nicholls and Ferguson, 2013). Decreased complex one oxidation of NADH and decreased respiration of mitochondria are both linked to drops in the NAD/NADH ratio (Titov et al., 2016). Complex II is the sole complex in ETC containing an immediate catalytic function in the Krebs cycle. It consists of a small protein complex containing four protein subunits made up of essential anchoring peptides and succinate dehydrogenase. The Fp and Ip subunits, composed of peptides containing three Fe-S or a flavin covalently linked, are split from the two big peptides that make up succinate dehydrogenase (Cadonic et al., 2016; Scheffler, 2007).

### 4.2 Complex III

Complex III’s structure is distinct from complex I and II because the structure is dimeric and made up of two identical chains of proteins. Every protein set has an identical arrangement of 11 subunits of protein and the Rieske Fe-S clusters cyt B 562, 566, and c1 metal redox centres. The Qo site, an attachment points for complex III that is located close to the intermembrane gap, is where complex III links QH_2_ produced in complex I and II. The Qi location, which is close to the mitochondrial matrix, is where Q is able to connect to complex III. On the other side of the Qo site, close to an intermembrane gap, cyt C is capable of binding to complex III, serving as complex III’s ultimate acceptor of electrons in redox processes (Cadonic et al., 2016; Scheffler, 2007). The ETCs last directly engaged complex in the flow of electrons is complex IV. Similar to complex III, complex IV is a big essential protein that is dimeric in form. Every portion of the protein consists of 13 chains of protein and several reductions of metal centres. These metal centres, which are divided into a bi-metallic CuA location, a monometallic Cyt A site, and a bi-metallic Cyt A3-CuB site, are predominantly composed of Fe and Cu. With the molecules of Cu attached by N-His, S-Met, S-Cys, and Glu residues in subunits of proteins within every monomer, CuA is located just beyond the membrane. Two N-His groups bind to cyt A to protein components, whereas four N-His clusters bind to cyt A3-CuB to the subunits of protein. In between both of these molecules is a small oxygen binding site; although the precise process of O_2_ reduction is not completely understood, molecular-level oxygen is the destination of electron transport in complex IV (Cadonic et al., 2016; Scheffler, 2007).

## 5 Mechanisms underlying mitochondrial dysfunction in AD and their associated therapies

The aetiology of multiple pathologies in AD remains elusive. The medications that have received approval from FDA for the treatment of AD are efficacious in their ability to address symptomatic therapy that affects neurotransmitters, specifically acetylcholine or glutamate (Table 1) (Cummings et al., 2024). The dysfunction of mitochondria during oxidative stress is widely recognized as a key characteristic of both acute and chronic neurodegenerative conditions, such as AD. Consequently, there has been growing interest in the development of mitochondria-targeted drugs as a promising therapeutic approach (Table 1) (Wang et al., 2025). It’s crucial to remember that this field of study is still in its infancy. The currently accessible treatment modalities can provide transient alleviation of symptoms, yet they are unable to decelerate the advancement of AD or effect a complete remedy (Yiannopoulou and Papageorgiou, 2020). In order to address this issue, there is an urgent need for transformative therapeutic interventions. Multiple targets are currently under investigation, and researchers are actively exploring novel methodologies in order to identify potential therapeutic alternatives. The present overview is centred on the latest knowledge pertaining to the paradigm of employing multitarget drugs for the treatment of AD in different phases of clinical trials and approved therapies.

**Table 1:** Overview of drugs investigated or used in Alzheimer’s disease therapy with their mechanistic basis.

S. no.	Drug	Mechanism of action	References
1	Donepezil	Reversibly inhibits acetylcholinesterase (AChE), preventing the breakdown of acetylcholine in synaptic clefts, thereby enhancing cholinergic neurotransmission and improving cognition. Also shown to promote non-amyloidogenic APP processing.	Zhang et al. (2020); Zhao et al. (2021)
2	Rivastigmine	Inhibits both acetylcholinesterase (AChE) and butyrylcholinesterase (BuChE), increasing acetylcholine levels in the cortex and hippocampus; indirectly stabilizes mitochondrial membrane potential via HIF-1α/VEGF signalling, improving neuronal survival.	Nguyen et al. (2021); Zhang et al. (2020)
3	Galantamine	Reversibly inhibits AChE and allosterically modulates nicotinic acetylcholine receptors (nAChRs), enhancing receptor sensitivity and acetylcholine release, which improves synaptic plasticity and memory function.	Lilienfeld (2002); Tundis et al. (2018)
4	Memantine	Non-competitive antagonist at NMDA-type glutamate receptors; blocks excessive Ca^2+^ influx through overactivated NMDA channels, preventing excitotoxic neuronal death, while allowing physiological neurotransmission involved in learning and memory.	Johnson and Kotermanski (2006); Parsons et al. (2013)
5	Aducanumab	Selectively binds to aggregated soluble oligomers and insoluble fibrillar Aβ plaques, promoting their clearance via microglia-mediated phagocytosis and reducing amyloid plaque burden, thereby slowing neurodegeneration.	Mukhopadhyay et al. (2021); Silvestro et al. (2022)
6	Lecanemab (Leqembi)	Targets soluble Aβ protofibrils (toxic oligomers) over insoluble plaques; enhances clearance of protofibrils from the brain and CSF, thereby reducing downstream tau pathology and slowing cognitive decline.	Niidome et al. (2024); Vitek et al. (2023)
7	Tacrine	Reversibly inhibits AChE, enhancing cholinergic neurotransmission; one of the first approved drugs but discontinued due to hepatotoxicity.	Mitra et al. (2022); Tian et al. (2021)
8	Gantenerumab	Binds to aggregated Aβ in parenchyma and vasculature, facilitating microglial-mediated clearance of plaques and potentially reducing neuroinflammation.	Miles and Masters (2025); Ostrowitzki et al. (2012)
9	Gosuranemab	Humanized IgG4 antibody that binds extracellular N-terminal tau fragments, preventing their spread between neurons and reducing tau-mediated neurotoxicity and synaptic dysfunction.	Parums (2021); Shulman et al. (2023)
10	Elenbecestat	Inhibits β-site APP cleaving enzyme 1 (BACE1), reducing the cleavage of amyloid precursor protein (APP) into Aβ peptides, thereby lowering Aβ production and aggregation.	Alan et al. (2023); Coimbra et al. (2024)

A neurodegenerative condition, AD, is characterized by the buildup of Aβ and NFTs; a rise in inflammatory proteins is also observed. Proteostasis, the coordinated regulation of protein synthesis, folding, trafficking, and degradation, is essential for neuronal health. Its collapse is increasingly recognized as a central contributor to AD pathogenesis, closely intersecting with mitochondrial dysfunction. Impairment of the ubiquitin–proteasome system and autophagy–lysosomal pathways lead to the accumulation of misfolded proteins such as Aβ and hyperphosphorylated tau, which further exacerbate mitochondrial stress (Llanos-González et al., 2020; Lu and Guo, 2020). Early downregulation of proteasome activity has been shown to precede overt neurodegeneration, suggesting proteostasis failure as an initiating event in AD progression (Jiang et al., 2025). Mitochondria serve as both regulators and victims of proteostasis imbalance. Oxidative stress arising from dysfunctional mitochondria damages chaperones and proteolytic enzymes, weakening the cellular ability to maintain protein homeostasis (Höhn et al., 2020; Trigo et al., 2023). Conversely, disrupted clearance of misfolded proteins interferes with mitochondrial protein import, dynamics, and quality control, creating a vicious cycle of energy failure and neuronal toxicity (Jagtap et al., 2023). Recent studies highlight that mitochondrial-associated proteostasis is particularly vulnerable in aging neurons, where inefficient stress responses accelerate neurodegeneration (Table 1) (Tseng et al., 2023). Importantly, loss of proteostasis not only amplifies Aβ and tau pathology but also impairs mitophagy and calcium signaling, linking it with other hallmarks of AD (Lu and Guo, 2020). These insights suggest that restoring proteostasis, through proteasome activation, chaperone upregulation, or enhancing mitochondrial stress responses, represents a promising therapeutic avenue for AD. Targeting this axis may help break the self-perpetuating cycle between mitochondrial dysfunction and protein aggregation.

Non-steroidal anti-inflammatory drugs (NSAIDs), according to epidemiological analyses, lower the chance of getting AD, which has sparked research on the impact of inflammation in AD. Mitophagy, the selective degradation of damaged mitochondria via autophagy, is a vital quality-control mechanism for maintaining neuronal homeostasis. Increasing evidence indicates that mitophagy is profoundly impaired in AD, leading to the accumulation of dysfunctional, ROS-producing mitochondria that aggravate synaptic and cognitive decline (Mary et al., 2023; Song et al., 2021). Key regulators such as PINK1 (PTEN-induced kinase 1) and Parkin, which normally orchestrate mitochondrial labeling and clearance, show reduced expression and activity in AD models and patient brains (Medala et al., 2021). Deficient mitophagy results in fragmented mitochondria persisting at synaptic terminals, impairing bioenergetics and neurotransmission. Aging further exacerbates mitophagy defects, as age-related declines in autophagic flux synergize with AD pathology to accelerate mitochondrial accumulation (Lyu et al., 2024; Song et al., 2021). Importantly, impaired mitophagy not only sustains oxidative stress but also disrupts calcium buffering and amplifies proteostasis failure, linking it with other key pathological processes in AD (Meng et al., 2025). Recent bibliometric analyses highlight mitophagy as one of the most rapidly emerging therapeutic targets in AD research (Wang et al., 2024). Pharmacological agents, gene therapy approaches, and natural compounds that restore mitophagic signaling have demonstrated neuroprotective effects in preclinical models, with improvements in synaptic plasticity and memory (Tang et al., 2025; Wang et al., 2025).

The suppression of cyclooxygenase (COX) is an extremely well-known effect of most NSAIDs (Hoozemans et al., 2008). In the conversion of arachidonic acid to prostanoids, specifically prostaglandins (PGs), COX serves as a restricting enzyme. PGs play a crucial role in neuroinflammation brought on by oxidative stress. The hallmarks of AD have been demonstrated to be somewhat improved by the administration of COX inhibitors, which include indomethacin, and the process is thought to entail a decrease in inflammation and oxidative stress. Celecoxib (a specific COX-2 inhibitor) also slows the development of the illness by reducing the death of neuronal cells due to its anti-inflammatory effects (El Halawany et al., 2017). The cytochrome oxidase enzyme is inhibited by an apolipoprotein E (ApoE) protein breakdown product that is targeted to mitochondria and has been identified in adult ApoE ϵ4 carriers’ postmortem brains. Since AD patients have reduced average cytochrome oxidase activities compared to age-matched control groups, A mechanistic relationship between ApoE ϵ4 and cytochrome oxidase appears to be especially interesting (Swerdlow, 2012; Wilkins et al., 2017). Although mtDNA contributes to the decline in AD cytochrome oxidase action, other moderators or prospective controllers of the function of mitochondria, including TOMM40 or ApoE, may also be involved (Wilkins et al., 2017).

COX-1 and COX-2 are two different variants of the COX enzyme that are typically linked to brain inflammation. While COX-2 has been elevated due to inflammasome activation within triggered microglia neurons, COX-1 is found in neurons of the brain’s cerebral cortex, along with the hippocampus. When neuroinflammation happens, inflammatory chemicals stimulate COX-2, which then makes and releases PGs through the arachidonic system and also stimulates microglia (Dhapola et al., 2021; Sil and Ghosh, 2016). Additionally, to be effective in reducing neuroinflammation in individuals with AD, COX-2 and iNOS suppression are needed (Bronzuoli et al., 2016; Dhapola et al., 2021). A research study is being conducted with celecoxib to avoid AD. Celecoxib decreased neuroinflammation in Aβ-induced rat models by decreasing the level of COX-2 proteins inside rat brains. NSAIDs reduce excessive expression of inflammatory mediators that cause neuroinflammation by selectively inhibiting COX-2 activities (Deardorff and Grossberg, 2017; Mhillaj et al., 2018). The production of cyt C oxidase IV, an enzyme found within ETC that helps produce ATP, is modulated in neurons by a brain-specific miRNA, miR338. Furthermore, the expression of miR338 is associated with COX-4 protein and mRNA levels (Aschrafi et al., 2012; Rivera et al., 2023) (Table 2).

**Table 2:** Consolidated overview of potential pharmacological interventions to date (now in clinical trials and also approved), elucidating their respective mechanisms of action and accompanying adverse effects.

S. no.	Drug	Class of drug	Approval year	Target pathways	Adverse effects	Clinical trial	References
1.	Amilomotide	Active immunotherapy (anti-Aβ vaccine)	Under clinical trials	Reducing Aβ production, preventing Aβ aggregation and promoting Aβ clearance	Abdominal pain, difficulty in breathing, irregular heartbeat, blurred vision	Phase II/III	Lopez Lopez et al. (2019); Song et al. (2022)
2.	Leqembi	Monoclonal antibody	2023	Targets soluble protofibrils (large oligomers) over plaques	Amyloid-Related Imaging Abnormalities	Phase IIb/III	Swanson et al. (2021)
3.	Aducanumab	Passive immunotherapy (anti-Aβ antibody)	2021	Targets and binds to aggregated soluble oligomers and insoluble fibril conformations of Aβ plaques	ARIA-edema, ARIA-hemosiderin deposition microhaemorrhage, Hypersensitivity	Phase IIIb	Dhillon (2021)
4.	Gantenerumab	Passive immunotherapy (anti-Aβ antibody)	2021	Interacts with aggregated brain Aβ, both parenchymal and vascular	Amyloid-Related Imaging Abnormalities (ARIAs)	Phase III	Ostrowitzki et al. (2018)
5.	Gosuranemab	Passive immunotherapy (IgG4 anti-tau)	2017	TAU inhibitors (Microtubule-associated protein tau inhibitors)	No adverse effects which lead to 100% decrease in unbound N-terminal tau fragments in CSF	Phase II	Dam et al. (2021)
6.	Elenbecestat	BACE1 inhibitor	2016	Reduction of plasma Aβ levels	Contact dermatitis, upper respiratory infection, headache, diarrhoea	Phase III	Plascencia-Villa and Perry (2020)
7.	Scyllo-inositol	Aβ aggregation Inhibitor	2009	Targets CDP-diacylglycerol-inositol 3-phosphatidyltransferase	Nausea, stomach pain, paraesthesia, headache, and dizziness	Phase II	Vaz and Silvestre (2020)
8.	Tideglusib	Thiadiazolidinone, GSK-3β inhibitor	2009	Increases the levels of neurotrophic peptide IGF-1, promotes endogenous hippocampal neurogenesis via GSK-3β inhibition	Transient increase in serum creatine kinase, ALT-or gGt-diarrhoea, nausea, cough, fatigue and headache	Phase II	Seneci (2015)
9.	Tramiprosate	Glycosaminoglycan (GAG)	2007	Binds to Lys16, Lys28, and Asp23 of Aβ42, thus reducing oligomeric and fibrillar (plaque) amyloid aggregation	Nausea, vomiting, and diarrhoea	Phase II	Manzano et al. (2020)
10.	Epothilone D	Macrolides, Microtubule stabilizer	2004	Inhibition of microtubule function by promoting axonal bud outburst, inhibiting the transition of the G1 phase to the S phase	Neutropenia and peripheral neuropathy	Phase II	Jeswani and Paul (2017)
11.	Memantine	NMDA Inhibitor	2003	p21, p38/MAPK and SAPK/JNK1/2 pathways	Dizziness, headache, confusion, diarrhoea, and constipation	Phase IIb	Kilic et al. (2013)
12.	Galantamine	Cholinesterase Inhibitor	2001	Prevention of the activation of P2X7 receptors, Protection of mitochondrial membrane potential, and Prevention of the membrane fluidity disturbances.	Inhibitory vagotonic effects on the cardiac conduction system, Bradycardia, Anesthesia, Gastrointestinal haemorrhage	Phase III	Tundis et al. (2018)
13.	Rivastigmine	Cholinesterase Inhibitor	2000	HIF-1α/VEGF signalling pathway	Gastrointestinal haemorrhage, angina and CNS symptoms	Phase III	Desai and Grossberg (2001)
14.	Tacrine	Cholinesterase Inhibitor	1993	Cholinergic Pathways	Hepatotoxicity	Phase III	Crismon (1994)
15.	Donepezil	Cholinesterase Inhibitor	1996	Enhancement of cholinergic neurotransmission, Promotion of non-amyloidodgenic pathways for APP processing	Gastrointestinal haemorrhage, Bradycardia, can also cause nightmares	Phase III	Abdelaal et al. (2017)

### 5.1 Therapeutic implications: targeting mitochondrial dysfunction in AD

Recent research has moved beyond traditional anti-amyloid strategies toward interventions that preserve mitochondrial integrity and cellular energetics. A growing body of clinical evidence underscores the translational potential of mitochondrial-directed therapeutics (Swerdlow, 2023; Tapia-Rojas and Inestrosa, 2018). Among these, MitoQ and SS-31 (elamipretide)—mitochondria-targeted antioxidants—have demonstrated efficacy in improving mitochondrial membrane potential, ATP synthesis, and reducing oxidative damage in preclinical AD models, with early-phase trials reporting favorable safety profiles and cognitive benefits (Cenini and Voos, 2019; Hemachandra Reddy et al., 2017). Similarly, nicotinamide riboside (NR), a precursor for NAD^+^ biosynthesis, enhances mitochondrial biogenesis and sirtuin activity, thereby improving neuronal resilience (Yusri et al., 2025). Urolithin A, derived from ellagitannins, promotes mitophagy and maintains synaptic function, showing promise in phase I/II trials for age-associated mitochondrial decline (Andreux et al., 2019; D’Amico et al., 2022). Additional compounds, such as coenzyme Q10 derivatives, spermidine, and metformin, are being evaluated for their capacity to modulate mitochondrial metabolism and oxidative stress in AD cohorts (Madeo et al., 2018; Zhou et al., 2018) (see Table 3).

**Table 3:** Recent clinical trials and emerging mitochondria-targeted therapeutics in Alzheimer’s disease.

Agent/class	Mechanism of action	Clinical status	Key findings	References
MitoQ	Mitochondria-targeted antioxidant; reduces ROS and lipid peroxidation	Phase II (completed)	Improved mitochondrial bioenergetics, mild cognitive benefit	Swerdlow (2023)
SS-31 (Elamipretide)	Cardiolipin stabilizer; enhances ETC function	Phase II (ongoing)	Improved mitochondrial coupling and ATP levels	Cenini and Voos (2019); Hemachandra Reddy et al. (2017)
Nicotinamide Riboside	NAD^+^ precursor; enhances mitochondrial biogenesis and sirtuin activity	Phase II	Improved cognitive and metabolic markers	Yusri et al. (2025)
Urolithin A	Mitophagy activator; promotes mitochondrial turnover	Phase I/II	Improved muscle and cognitive function in aging models	Andreux et al. (2019); D’Amico et al. (2022)
Coenzyme Q10 derivatives	ETC cofactor; antioxidant	Phase II	Mixed outcomes; limited CNS penetration	Mantle and Hargreaves (2025)
Spermidine	Autophagy and mitophagy enhancer	Phase I	Improved mitochondrial clearance and synaptic markers	Madeo et al. (2018)
Metformin	AMPK activator; modulates mitochondrial metabolism	Phase II (repurposed)	Improved glucose and energy metabolism; under evaluation for cognitive outcomes	Zhou et al. (2018)

## 6 COX-1/2/3 and their link up to AD: mechanistic overview

In AD, COX-1 and COX-2 expression levels change across disease stages. Early on, COX-2 rises in neurons alongside low fibrillar Aβ and limited NFTs, colocalizing with cell cycle enzymes, while COX-1 is mainly produced in microglia near fibrillar Aβ deposits (Hoozemans et al., 2008). This indicates COX-1/2 roles in inflammatory and regenerative networks of the AD brain. However, most studies show NSAIDs or COX-2 inhibitors fail to protect against AD, highlighting their pathogenic rather than protective involvement (Hoozemans et al., 2008). COX-2 is also linked to abnormal cell cycle protein expression and regeneration processes. RNA microarray studies confirm early upregulation of genes related to proliferation, differentiation, adhesion and PG synthesis (Blalock et al., 2004; Hoozemans et al., 2004, 2005). These findings motivated trials with anti-inflammatory drugs targeting COX-2 (McGeer and McGeer, 2007).

COX, a membrane-bound glycoprotein, converts arachidonic acid into prostanoids such as PGE2, PGF2-*α*, PGD2, PGI2, and thromboxane (TX) A2. Two isoforms exist: COX-1 on Chr 9 and COX-2 on Chr 1. COX-1 is constitutively expressed and maintains physiological functions such as renal and gastric protection, while COX-2 has a TATA box and multiple transcription factor sites enabling complex regulation by hormones, cytokines and endotoxins (Hoozemans et al., 2008). Though structurally similar, slight catalytic region differences allow isoform-specific inhibitors. Because of cardiovascular risks, COX-2 inhibitor trials were discontinued. Both isoforms are constitutively expressed in neocortex and hippocampus, supporting brain function. COX-2 and PGE2 also regulate synaptic plasticity and excitability (Chen et al., 2002), while COX-2 is strongly induced in inflammatory sites (Vane et al., 1998). In AD, COX-2 is upregulated in activated microglia and astrocytes, and strongly expressed in neurons, especially pyramidal cells vulnerable in moderate AD. Early susceptibility stages show elevated neuronal COX-2, though its exact role in pathogenesis remains unclear. Since COX-2 is regulated by synaptic activity, its early increase likely reflects heightened neuronal activity, while later reductions may result from declining synaptic function and neuronal loss (Yermakova and Kerry O’Banion, 2001).

COX-1 positive microglia cluster around conventional and neuronal plaques when Aβ and COX-1 are double immunostained. Major Histocompatibility Complex (MHC) class 2 levels do not affect COX-1 expression, indicating microglial activation does not raise COX-1, which functions constitutively in mature rat and human microglia (Hoozemans et al., 2002). Triflusal, a salicylic acid derivative and irreversible COX-1/platelet aggregation inhibitor, improved cognition and axonal curvatures deficits in AD transgenic mice (Coma et al., 2010). Longer treatments reduced plaque load, glial activation, pro-inflammatory cytokines and neurodegeneration markers, similar to other NSAIDs (Choi et al., 2010). In contrast, COX-2 inhibitors like rofecoxib failed in MCI or AD and even increased AD risk in MCI trials (Aisen et al., 2008). COX-2 rises early in AD, and pre-MCI trials with the COX-2 inhibitor celecoxib showed cognitive benefits with minimal metabolic deficits by fluorodeoxyglucose positron emission tomography (FDG-PET). COX-2 inhibitors may correct early LTP and cognition defects, whereas COX-1 and 2 appear to act at different AD stages—COX-2 inhibitors are more useful pre-MCI, while COX-1 antagonism is unlikely to be beneficial (Kotilinek et al., 2008; Small et al., 2008). However, COX-2 inhibitors suppress vasodilatory PGI2 in endothelium, raising TX levels and platelet aggregation (Jespersen et al., 2009). Highly selective COX-1/2 inhibition can also trigger arachidonic acid accumulation, linked to AD, so they may m not be ideal anti-inflammatory targets (Ikonomovic et al., 2008). COX-1 may contribute to neuroinflammation and cognitive decline but not Aβ pathology, and triflusal showed safety and benefits in animal and clinical studies (Frautschy, 2010).

COX-3, a COX-1 variant sensitive to paracetamol, has been studied with AD in (Interleukin (IL)-1β + Aβ42)-stimulated human neuronal cultures, as COX-2 is elevated in AD while COX-3 is enriched in mammalian CNS (Warner and Mitchell, 2002; Cui et al., 2004). COX-3 arises from COX-1 via retention of intron 1, forming a distinct RNA transcript (Chandrasekharan et al., 2002). It is thought to be therapeutically distinct from COX-1/2, being selectively inhibited by acetaminophen. COX-2 signalling is elevated in AD brains, and COX-3 is abundant in the cerebral and temporal cortex (Bazan and Lukiw, 2002; Xiang et al., 2002). COX-3 likely coexists with COX-1 in AD affected regions, possibly influenced by stem-loop structures in COX-1 intron (Cui et al., 2004). COX-3 and COX-1 RNAs have similar half-lives (~12 h) and may serve auxiliary nuclear or membrane cyclooxygenation roles (Bazan and Lukiw, 2002). Many consider COX-3 an altered COX-1 with distinct pharmacology (Phillis et al., 2006). Analgesics like acetaminophen, phenacetin, antipyrine, and dipyrone, and several NSAIDs inhibit COX-3, likely mediating their antipyrine effects (Chandrasekharan et al., 2002; Phillis et al., 2006). COX-3 mRNA is highest in choroid plexus and spinal cord, then the hypothalamus, hippocampus, medulla, cerebellum, cerebral vessels and cortex, especially endothelial cells (Kis et al., 2004). When introduced into COX-deficient COS (CV-1 in Origin)-7 cells, COX-3 lacked PG synthesis activity (Figure 3) (Snipes et al., 2005). It may support membrane COX signalling during basal COX-1/2 expression (Yagami et al., 2016) (Figure 3).

## 7 Key player: a role of cardiolipin in AD progression

Cardiolipin (CL) is an anionic phospholipid with a distinctive double glycerophosphate core and four lipid side chains. It predominantly resides in the inner mitochondrial membrane, where it plays essential roles in maintaining membrane structure, stability, and activity, while also being present in peripheral tissues (Li et al., 2015; Pointer and Klegeris, 2017). Within the CNS, CL is found in both neuronal and non-neuronal glial cells, where it regulates metabolic and mitochondrial processes to support brain cell survival (Mejia et al., 2014). Multiple molecular forms of CL exist in the CNS, promoting mitochondrial integrity and neuronal longevity (Cheng et al., 2008). CL sustains ETC efficiency, mitochondrial fusion and fission, and intracellular signaling. By stabilizing complexes I, II, and IV into supercomplexes, CL enhances electron transfer, reduces ROS generation, and promotes an electrochemical gradient across the inner membrane (Cui et al., 2014; Pointer and Klegeris, 2017).

Alterations in CL composition or levels lead to dysfunctional, swollen mitochondria, contributing to neurodegenerative disorders such as AD and PD, and worsening outcomes after traumatic brain injury (Ghio et al., 2016; Ji et al., 2012; Sathappa and Alder, 2016). CL-containing liposomes are being explored as potential therapeutic carriers for drug delivery in AD, possibly aiding in Aβ clearance or plaque reduction (Orlando et al., 2013). Structurally, CL is a non-bilayer-forming glycerophospholipid with a conical shape due to its four fatty acyl chains, which is critical for mitochondrial architecture (Falabella et al., 2021; Schlame and Ren, 2009). It is mainly localized in the inner mitochondrial membrane (IMM), adjacent to OXPHOS proteins. Defective CL biosynthesis alters mitochondrial morphology and dynamics, impairing OXPHOS and energy adaptation (Kasahara et al., 2020; Sustarsic et al., 2018; Zhang et al., 2011). CL also supports the assembly of translocase complexes TOM and TIM23; its deficiency hampers protein import and disrupts mitochondrial function (Malhotra et al., 2017). Furthermore, CL is required for the optimal activity of several transporters, including carnitine/acylcarnitine, phosphate, pyruvate, tricarboxylate carriers, and calcium uniporters (Ghosh et al., 2020; Senoo et al., 2020). In AD animal models, decreased CL levels correlate with synaptic mitochondrial dysfunction and elevated oxidative stress, suggesting its role in disease progression (Monteiro-Cardoso et al., 2014). Tau preferentially binds to CL-rich regions of the outer mitochondrial membrane, inducing swelling, cytochrome c release, and loss of membrane potential. Both *in vivo* and *in vitro* studies demonstrate that CL-rich mitochondrial membranes are particularly vulnerable to tau aggregates, promoting neuronal toxicity (Camilleri et al., 2020; Cheng and Bai, 2018).

## 8 Mitochondrial free radical production and longevity (MFRTA)

The mitochondrial free radical theory of ageing (MFRTA) (Son and Lee, 2021) emerged in 1972 as an alternative to the wear-and-tear and antioxidant-based concepts of ageing. Early studies focused on manipulating whole-cell and tissue antioxidants to slow ageing by enhancing antioxidant availability. This focus stemmed from the relative ease of controlling antioxidant levels compared to mitochondrial ROS production (mtROSp), which remained technically challenging to measure for decades. During the 1970s and 1980s, numerous rodent studies manipulating antioxidant systems consistently demonstrated that increasing enzymatic or non-enzymatic antioxidants, even through dietary supplementation or genetic amplification, did not extend lifespan (Barja, 2004b, 2004a). The only exception was a study in which enhanced catalytic activity within mitochondria, but not peroxisomes or the nucleus, led to increased longevity (Schriner et al., 2005). Comparative analyses of antioxidant levels across species also revealed no positive correlation with lifespan; instead, 21 of 27 associations reported across five independent laboratories were negative (Perez-Campo et al., 1998).

It was later proposed that mtROSp decreases with increased species longevity, a hypothesis supported by cross-species evidence. Birds, for instance, exhibit lower mtROSp than mammals of comparable body size and metabolic rate, contributing to their longer lifespans. Pigeons, parakeets, and canaries have significantly reduced mtROSp relative to rodents, corresponding to lifespans of 30, 20, and 24 years respectively, despite comparable oxygen consumption and electron transport efficiency (Gómez et al., 2023). These findings indicate that mtROSp and free radical leak (FRL) are not fixed proportions of electron flux but are actively regulated, suggesting that organisms can modulate mtROSp to influence longevity without altering mitochondrial respiration or aerobic metabolism. Baseline mtROSp levels, typically <1% of total electron flux, are controlled by nuclear-encoded factors that fine-tune the expression of mitochondrial components generating ROS, often through transcriptional regulators such as Forkhead box proteins (FOXOs). Consequently, each species exhibits a characteristic mtROSp level optimized for its evolutionary niche and lifespan (Brunet-Rossinni and Austad, 2004).

## 9 Pro-longevity interventions associated with mitochondria

The role of mtROSp as an activator of ageing is not limited to interspecies variations. Caloric restriction (CR), the most well-established lifespan-extending intervention, can increase mammalian longevity by up to 40% while reducing mtROSp, FRL, and 8-oxodG levels in mitochondrial DNA by a similar 30%–40% (Austad and Hoffman, 2021). These proportional decreases correspond to roughly a 1.4-fold increase in lifespan. Interestingly, all other known lifespan-extending interventions in mammals, such as protein restriction (PR), rapamycin administration, and long-lived single-gene mutants, also reach this 1.4-fold threshold. However, interspecies differences can exceed 10-fold, suggesting additional ageing mechanisms beyond oxidative stress regulation. The Unified Theory of Ageing proposes that multiple ageing pathways, in addition to mtROSp, influence lifespan variation across species (Barja, 2019).

Rodent longevity is similarly enhanced by PR and, more specifically, by dietary methionine (Met) restriction. Limiting Met intake prolongs life in rats and mice (Fang et al., 2022) and lowers mtROSp, FRL, and 8-oxodG in mtDNA to the same extent as CR, though CR doubles lifespan relative to Met restriction. In contrast, restricting other amino acids does not reduce oxidative markers, suggesting that Met limitation is the main driver of CR’s antioxidant benefits (Pamplona and Barja, 2006; Sanchez-Roman and Barja, 2013). Conversely, excessive Met intake elevates oxidative stress and mtROSp, consistent with the high protein consumption common in developed nations, which often exceeds dietary recommendations by 2-3 times (Żebrowska et al., 2019). Elevated Met levels impair circulatory and hepatic function, increasing iron load and metabolites such as S-adenosylmethionine (SAM), S-adenosylhomocysteine (SAH), cysteine, and homocysteine (Kumagai et al., 2002; Park et al., 2008). Met-induced oxidative stress also promotes protein methionine oxidation to methionine sulfoxide (MetSO), reducing protein activity, whereas enhanced MetSO reductase expression in long-lived *Drosophila melanogaster* repairs MetSO and protects against oxidative damage (Lim et al., 2012; Uthus and Brown-Borg, 2006).

There is strong evidence that excess dietary Met exerts direct pro-oxidant effects. In isolated mitochondria, Met supplementation increases mtROSp in liver and kidney tissues (Gomez et al., 2011; Troen et al., 2003), suggesting a direct mitochondrial interaction. Elevated Met intake raises circulating SAM, SAH, and homocysteine levels in both rats and humans (Gomez et al., 2009; Velez-Carrasco et al., 2008), metabolites linked to atherosclerosis, hypertension, and vascular and cognitive ageing (Elshorbagy et al., 2008; Troen et al., 2003). Furthermore, dietary cysteine supplementation can counteract many adverse effects of Met, including elevated mtROSp, mtDNA damage, weight gain, adiposity, and related hormonal imbalances (Elshorbagy et al., 2011; Gomez et al., 2015).

## 10 Future prospective

While the past decades of research have significantly advanced our understanding of AD, the absence of curative therapies underscores an urgent need to redefine our approach. Given the central role of mitochondria in orchestrating neuronal metabolism, redox balance, and synaptic function, future studies should prioritize interventions that restore mitochondrial homeostasis. Strategies enhancing mitophagy and mitochondrial biogenesis, improving mitochondrial dynamics (fusion/fission), and stabilizing the ETC are promising avenues that require rigorous validation through preclinical and clinical studies. Emerging research on mitochondrial proteostasis and inter-organelle communication (particularly ER–mitochondria crosstalk) further suggests that maintaining protein quality control is essential for neuronal resilience and therapeutic success. Moreover, integrating multi-omics platforms (genomics, transcriptomics, proteomics, metabolomics) could help identify reliable early biomarkers of mitochondrial dysfunction and therapeutic response, enabling patient stratification and personalized treatment regimens. Combining mitochondrial-directed interventions with synaptic repair approaches, anti-amyloid and anti-tau strategies, and neuroinflammation modulators may yield synergistic effects that surpass monotherapies. Advanced drug delivery systems such as nanocarriers, exosome-based vehicles, and blood–brain barrier-penetrating peptides hold potential to improve the CNS bioavailability of mitochondria-targeted drugs. Artificial intelligence and machine learning could further accelerate therapeutic discovery by predicting drug–target interactions and optimizing clinical trial designs. Additionally, repurposing FDA-approved drugs with known mitochondrial modulatory effects may shorten the translational timeline. Hence, integrating these multidisciplinary strategies holds promise for transforming mitochondrial research into precision therapeutics capable of modifying AD progression.

## 11 Conclusion

AD is a multifactorial neurodegenerative disorder in which mitochondrial dysfunction emerges as a pivotal upstream driver of pathological cascades, including oxidative stress, calcium dyshomeostasis, proteostasis failure, mitophagy impairment, and synaptic degeneration. Current treatments offer only transient symptomatic relief and fail to prevent disease progression, largely because they target downstream manifestations rather than initiating events. This review synthesizes evidence implicating mitochondrial dysfunction as a central hub that links diverse pathological mechanisms in AD. Correcting mitochondrial impairments could attenuate oxidative stress, preserve synaptic integrity, restore neuronal energy metabolism, and ultimately delay neurodegeneration. While significant progress has been made in delineating these pathways, substantial gaps remain in translating this knowledge into effective clinical interventions. A multitarget therapeutic approach, combining agents that simultaneously modulate mitochondrial function, reduce Aβ and tau pathology, and restore synaptic homeostasis, may hold the greatest promise. Bridging basic mechanistic research with translational and clinical efforts will be crucial. Strengthening interdisciplinary collaborations between neuroscientists, mitochondrial biologists, pharmacologists, and bioengineers will accelerate this progress. Hence, focusing on mitochondria as a unifying therapeutic axis represents a promising frontier in AD research. Advancing this line of investigation could pave the way for the development of disease-modifying therapies that move beyond symptomatic relief and truly alter the trajectory of AD.

## Glossary

AD: Alzheimer’s Disease
ApoE: Apolipoprotein E
APP: Amyloid Precursor Protein
ATP: Adenosine Triphosphate
Aβ: Amyloid β
BACE1: Beta-APP-Cleaving Enzyme-1
Ca^2+^: Calcium Signalling
Chr: Chromosome
CL: Cardiolipin
CNS: Central Nervous System
COS: CV-1 in Origin
COX: Cyclooxygenase
CR: Caloric Restriction
Cyt: Cytochrome
EOAD: Early Onset of AD
ETC: Electron Transport Chain
FDG-PET: Fluorodeoxyglucose Positron Emission Tomography.
FMN: Flavin Mononucleotide
FOXO: Forkhead box protein
FRL: Free Radical Leak
IL: Interleukin
ISR: Integrated Stress Response
LOAD: Late Onset of AD
LTD: Long Term Depression
LTP: Long Term Potentiation
MCI: Mild Cognitive Impairment
Met: Methionine
MFRTA: Mitochondrial Free Radical Theory of Aging
MHC: Major Histocompatibility Complex
miRNA: microRNA
mtDNA: mitochondrial DNA
mtROSp: mitochondrial ROS production
MTs: Microtubules
mtUPR: mitochondrial Unfolded Protein Response
NFTs: Neurofibrillary Tangles
NSAID: Non-Steroidal Anti-Inflammatory Drugs
OMM/IMM: Outer/Inner Mitochondrial Membrane
OXPHOS: Oxidative Phosphorylation
PGs: Prostaglandins
PHFs: Paired Helical Filaments
PINK1: PTEN-induced kinase 1
PR: Protein Restriction
RAGE: Receptor for Advanced Glycosylation End products
ROS: Reactive Oxygen Species
SAH: S-adenosylhomocysteine
SAM: S-adenosylmethionine
SVs: Synaptic Vesicles
TX: Thromboxane
